# Statistical models versus machine learning for competing risks: development and validation of prognostic models

**DOI:** 10.1186/s12874-023-01866-z

**Published:** 2023-02-24

**Authors:** Georgios Kantidakis, Hein Putter, Saskia Litière, Marta Fiocco

**Affiliations:** 1 Mathematical Institute (MI) Leiden University, Niels Bohrweg 1, 2333 CA Leiden, The Netherlands; 2 Department of Biomedical Data Sciences, Section Medical Statistics, Leiden University Medical Center (LUMC), Albinusdreef 2, 2333 ZA Leiden, The Netherlands; 3 Department of Statistics, European Organisation for Research and Treatment of Cancer (EORTC) Headquarters, Ave E. Mounier 83/11, 1200 Brussels, Belgium; 4 Trial and Data Center, Princess Máxima Center for pediatric oncology (PMC), Heidelberglaan 25, 3584 CS Utrecht, the Netherlands

**Keywords:** Artificial neural networks, Competing risks, Predictive performance, Random survival forests, Regression models, Supervised machine learning, Survival analysis

## Abstract

**Background:**

In health research, several chronic diseases are susceptible to competing risks (CRs). Initially, statistical models (SM) were developed to estimate the cumulative incidence of an event in the presence of CRs. As recently there is a growing interest in applying machine learning (ML) for clinical prediction, these techniques have also been extended to model CRs but literature is limited. Here, our aim is to investigate the potential role of ML versus SM for CRs within non-complex data (small/medium sample size, low dimensional setting).

**Methods:**

A dataset with 3826 retrospectively collected patients with extremity soft-tissue sarcoma (eSTS) and nine predictors is used to evaluate model-predictive performance in terms of discrimination and calibration. Two SM (cause-specific Cox, Fine-Gray) and three ML techniques are compared for CRs in a simple clinical setting. ML models include an original partial logistic artificial neural network for CRs (PLANNCR original), a PLANNCR with novel specifications in terms of architecture (PLANNCR extended), and a random survival forest for CRs (RSFCR). The clinical endpoint is the time in years between surgery and disease progression (event of interest) or death (competing event). Time points of interest are 2, 5, and 10 years.

**Results:**

Based on the original eSTS data, 100 bootstrapped training datasets are drawn. Performance of the final models is assessed on validation data (left out samples) by employing as measures the Brier score and the Area Under the Curve (AUC) with CRs. Miscalibration (absolute accuracy error) is also estimated. Results show that the ML models are able to reach a comparable performance versus the SM at 2, 5, and 10 years regarding both Brier score and AUC (95% confidence intervals overlapped). However, the SM are frequently better calibrated.

**Conclusions:**

Overall, ML techniques are less practical as they require substantial implementation time (data preprocessing, hyperparameter tuning, computational intensity), whereas regression methods can perform well without the additional workload of model training. As such, for non-complex real life survival data, these techniques should only be applied complementary to SM as exploratory tools of model’s performance. More attention to model calibration is urgently needed.

**Supplementary Information:**

The online version contains supplementary material available at 10.1186/s12874-023-01866-z.

## Background

Survival analysis (also referred as time-to-event analysis) is used to estimate the lifespan of a particular population under study. Frequently, survival data are right censored; time to event is not observed for all patients due to follow-up interruption before experiencing the event of interest or time limitations (study termination). Competing risks (CRs) occur frequently in clinical applications of survival data [1–4]. In this type of data an individual may fail from one of several causes. A CR is an event whose occurrence precludes the occurrence of an event of interest (for instance death may preclude the occurrence of disease relapse) [5, 6]. In health research, CRs are unlikely to be independent as the biology suggests at least some dependence between events. In several chronic diseases attributable to aging and frailty such as cancer, chronic heart failure, or dementia, study populations are susceptible to CRs [7].

The most popular non-parametric approach to estimate survival in the presence of right censored time-to-event data is the Kaplan-Meier’s methodology (KM) [8]. However, in the presence of CRs, this methodology overestimates the probability of failure which might lead to over-treatment of patients [1, 5, 9]. Different statistical models (SM) have been developed to estimate the cumulative incidence (absolute risk) of an event in the presence of CRs such as the cause-specific Cox model [10], and the Fine-Gray sub-distribution hazards regression model [11]. The former is a natural extension of the standard proportional hazards Cox model for the CRs setting where a Cox model is applied for each cause-specific hazard. The latter models the effect of covariates directly on the cumulative incidence function (CIF) over time reporting on the sub-distribution hazard ratio [9].

Nowadays, there is a growing interest in applying machine learning (ML) for prediction (diagnosis or prognosis) of clinical outcomes [12, 13] which has sparked a debate regarding the added value of ML techniques versus SM in the medical field. Criticism is attributed to ML prediction models. Despite no assumptions about the data structure are made, and being able to naturally incorporate interactions between predictive features, they are prone to overfitting of the training data and they lack extensive assessment of predictive accuracy (i.e., absence of calibration curves) [14, 15]. On the other hand, traditional regression methods are considered straightforward to use and harder to overfit. That being said, they do make certain (usually strong) assumptions such as the proportional hazards over time for the Cox model, and require manual pre-specification of interaction terms.

Amongst ML techniques, artificial neural networks have been a common choice in healthcare. This trend is pertinent with the collection of large and complex patient information in electronic health records, and the rise of computational power [16]. Over the years, neural networks and other ML techniques have been developed for survival data. Wang et al. in 2019 provide a comprehensive survey of conventional and modern approaches for right-censored time-to-event data [17]. The authors describe several ML techniques and suggest that neural networks are well-suited to predict survival and estimate disease risk.

A common approach in the literature is the partial logistic artificial neural network (PLANN) of Biganzoli et al. (1998) [18]. For the purpose of implementation, time is specified in discrete non-overlapping time intervals which are added as an input feature in a longitudinally transformed feed-forward network with logistic activation, and entropy error function. The output layer estimates smoothed discrete hazards for each time interval. PLANN was extended by Lisboa et al. (2003) under a Bayesian regularisation framework which performs automatic relevance determination (PLANN-ARD) [19]. Recently, Kantidakis et al. in 2020 proposed extensions of PLANN in terms of architecture i.e., new hyperparameters, new activation functions, and time interval specification as multiple input features [20]. Next to survival neural networks (SNNs), another well-known ML technique for clinical prediction of survival data is random survival forests (RSF, Ishwaran et al. 2008) [21]. RSF adapt Breiman’s random forest method by using a collection of survival trees [22].

ML approaches have also been employed for CRs, but the literature is limited. The PLANNCR approach was developed by Biganzoli et al. in 2006 for the joint modelling of discrete cause-specific hazards [23]. This extends PLANN by using the time (in discrete time intervals) as an input feature in a longitudinally transformed network with multinomial error function and logistic - softmax activation functions for the hidden and the output layer (multiple output nodes), respectively. Later, Lisboa et al. (2009) implemented PLANNCR under a Bayesian regularisation framework (PLANNCR-ARD) [24]. Ishwaran et al. extended RSF for CRs (RSFCR) in 2014 to estimate the CIF of competing events [25].

For this work, a dataset with small/medium sample size and limited number of predictive features (low-dimensional setting) is analysed. This concerns a retrospectively collected cohort of 3826 patients with high-grade extremity soft-tissue sarcomas (eSTS) treated surgically with curative intent. Nine prognostic factors are used to develop and validate several clinical prediction models with CRs for ML techniques and SM. The clinical endpoint of the study is defined as the time in years between surgery and disease progression (as local recurrence or distant metastasis; event of interest) of eSTS, where death is a competing event. Time points of interest are 2, 5, and 10 years (5-year horizon is of major clinical interest). Analyses were performed in R programming language version 4.1.2 [26].

The aims of this manuscript can be summarised as: (i) examination of extensions of PLANNCR method (PLANNCR extended) for the development and validation of prognostic clinical prediction models with competing events, (ii) systematic evaluation of model-predictive performance for ML techniques (PLANNCR original, PLANNCR extended, RSFCR) and SM (cause-specific Cox, Fine-Gray) regarding discrimination and calibration, (iii) investigation of the potential role of ML in contrast to conventional regression methods for CRs in non-complex eSTS data (small/medium sample size, low dimensional setting), (iv) practical utility of the methods for prediction.

The paper is organized as follows. In Section “Methods”, the eSTS data is presented. Further sections discuss basic concepts for CRs, the SM and the ML techniques, model training, and how the predictive performance was assessed. Section “Results” describes PLANNCR extended tuned with two measures, and compares the predictive performance of all methods in terms of discrimination and calibration. The manuscript ends with a “Discussion” about findings, limitations, and future perspectives of this work.

## Methods

This section is divided into several subsections where the methodology used for this work is presented to the reader. To begin with, the clinical data is described. Next, the SM and the ML techniques are discussed. Two well-known statistical models for CRs are employed: the cause-specific Cox model [10], and the Fine-Gray sub-distribution hazards regression model [11], as well as two extensions of popular ML techniques for CRs: the RSFCR [25], and the PLANNCR [23] as originally developed or with some modifications. Afterwards, it is presented how the models were trained, and which performance measures were used to evaluate their predictive ability. More technical details are provided in the Supplementary material.

### Dataset

Extremity soft-tissue sarcomas (eSTS) constitute a wide variety of histological subtypes with different sizes and grades that affect patients of any age group. Treatment protocols may differ between institutes and countries. Hence, important differences can be observed in the clinical course and prognosis of patients [27]. Over the years, several prognostic prediction models have been developed for overall survival and local recurrence [28–30].

For this project, a retrospectively collected cohort of 3826 patients with eSTS was used [29]. The dataset contained pseudo-anonymised patients from Leiden University Medical Center (Leiden, the Netherlands), Royal Orthopaedic Hospital (Birmingham and Stanmore, UK), Netherlands Cancer Institute (Amsterdam, the Netherlands), Mount Sinai Hospital (Toronto, Canada), the Norwegian Radium Hospital (Oslo, Norway), Aarhus University Hospital (Aarhus, Denmark), Skåne University Hospital (Lund, Sweden), Medical University Graz (Graz, Austria), Royal Marsden Hospital (London, UK), Daniel den Hoed (Rotterdam, the Netherlands), Radboud University Medical Center (Nijmegen, the Netherlands), University Medical Center Groningen (Groningen, the Netherlands), Haukeland University Hospital (Bergen, Norway), Helios Klinikum Berlin-Buch (Berlin, Germany), MedUni Vienna (Vienna, Austria), Vienna General Hospital (Vienna, Austria). In addition, eSTS patients from EORTC 62931 randomised controlled trial were included [31]. Data from the centers was collected between January 2000 and December 2014. Patients from the EORTC trial were recruited between February 1995 and December 2003.

**Table 1:** Patient demographics. sd, standard deviation; $R_{0}$, negative margin; $R_{1-2}$, positive margin with tumor cells in the inked surface of the resection margin; MFH/UPS/NOS, alignant fibrous histiocytoma / undifferentiated pleomorphic sarcoma / (pleomorphic) soft tissue sarcomas not-otherwise-specified; histology “Other”, angiosarcoma, clear cell sarcoma, conventional fibrosarcoma, epithelioid sarcoma, giant cell sarcoma, malignant granular cell tumor, malignant peripheral nerve sheath tumor, rhabdomyosarcoma (adult form), spindle cell sarcoma, unclassified soft tissue sarcoma and undifferentiated sarcoma

Characteristics	Total (N = 3826)
Gender (%)	
Female	1713 (44.77%)
Male	2113 (55.23%)
Mean age in years (sd)	59.40 (18.04)
Mean tumor size in cm (sd)	8.97 (5.69)
Surgical margin (%)	
$R_{0}$	3310 (86.51%)
$R_{1-2}$	516 (13.49%)
Adjuvant chemotherapy (%)	
No	3350 (87.56%)
Yes	476 (12.44%)
Tumor grade (%)	
II	656 (17.15%)
III	3170 (82.85%)
Histological subtype (%)	
Myxofibrosarcoma	771 (20.15%)
Synovial sarcoma	450 (11.76%)
MFH/UPS/NOS	1330 (34.76%)
Leiomyosarcoma	385 (10.06%)
Liposarcoma	421 (11.00%)
Other	469 (12.26%)
Tumor depth (%)	
Superficial	1014 (26.50%)
Deep	2812 (73.50%)
Radiotherapy (%)	
No	1341 (35.05%)
Neoadjuvant	521 (13.62%)
Adjuvant	1964 (51.13%)

Patients were selected from the sarcoma registry of each hospital based on histological diagnosis. Those initially treated without curative intent, showed local recurrence or distant metastasis at baseline, had Kaposi’s sarcoma or rhabdomyosarcoma (pediatric form), tumor was present in their abdomen, thorax, head or neck, or were treated with isolated limp perfusion as neoadjuvant treatment were excluded from the collection.

The dataset contained nine prognostic factors. Seven were categorical; *gender* (female or male), *surgical margin* ($R_{0}$ for negative or $R_{1-2}$ for positive with tumor cells in the inked surface of the resection margin), *adjuvant chemotherapy* (no or yes), *tumor grade* (II or III), *tumor depth* in relation to investing fascia (superficial or deep), *radiotherapy* (no, neoadjuvant or adjuvant), *histological subtype* (myxofibrosarcoma, synovial sarcoma, malignant fibrous histiocytoma / undifferentiated pleomorphic sarcoma / (pleomorphic) soft tissue sarcomas not-otherwise-specified, leiomyosarcoma, liposarcoma or other). Two were continuous; *age* at baseline (in years) and *tumor size* by the largest diameter measured at pathological examination (in centimetres).

Median follow-up survival time is 5.98 years estimated by reverse Kaplan-Meier (25% quartile: 3.94 years, 75% quartile: 8.80 years, range: 0.01 to 16.85 years) [8]. The endpoint of interest is defined as the time in years between surgery and disease progression (local recurrence or distant metastasis) of eSTS, with death as competing event; 1773 patients were alive/censored at the end of follow-up (46.34%), 1554 had disease progression (40.62%), and 499 died without local recurrence/distant metastasis (13.04%).

The dataset contained 3.70% missing data overall for the nine variables, with 2514 complete cases (65.71%). More specifically, there were missing values (0.97-11%) for all variables; 11.00% for *tumor depth* (421/3826), 8.21% for *histological subtype* (314/3826), 7.40% for *surgical margin* (283/3826), 4.36% for *adjuvant chemotherapy* (167/3826), 4.05% for *tumor size* (155/3826), 3.53% for *gender* (135/3826), 2.61% for *radiotherapy* (100/3826), 1.99% for *tumor grade* (76/3826), and 0.97% for *age* (37/3826), in decreasing order, respectively.

A simple imputation was used to avoid discarding observations from nearly complete records. The missForest algorithm was applied to reconstruct any missing values, which is the most exhaustive/accurate random forest algorithm for missing data [32]. This is a nonparametric imputation method that does not make any a priory assumptions regarding the data structure. A random forest with 1000 trees (for model stability) was built for each variable with missing information, testing all possible variable combinations as responses. Table 1 provides patient demographics of the final dataset (demographics of the original dataset are provided in Table S1 of Additional file 1).

### Basic concepts for competing risks

Typically for survival data, if several types of events occur, a model describing progression for each of the CRs is needed. The observable data is represented by the time of failure *T*, the cause of failure *D* ($D \in 1, \cdots , k$, $k \ge 1$; here *k* = 2), and a covariate vector $\mathbf {Z}$. Usually there is one type of event that is of interest (i.e., disease progression as local recurrence or distant metastasis) whereas the other events could prevent it from occurring (here competing event is death).

Following Putter et al. (2007) [1], a fundamental concept in modelling CRs is the cause-specific hazard function which denotes the hazard of failing from a given cause in the presence of CRs:

1 $$\begin{aligned} \lambda _{k}(t) = {\underset{\Delta t \rightarrow 0}{\lim}} \frac{ Prob(t \leq T < t + \Delta t, D = k | T \geq t )}{\Delta t}. \end{aligned}$$

Then, the cumulative cause-specific hazard can be specified as

2 $$\begin{aligned} \Lambda _{k}(t) = \int _{0}^{t} \lambda _{k}(s) ds \end{aligned}$$

and the survival function (probability of not having failed from any cause at time t) can be written as

3 $$\begin{aligned} S(t) = \exp {\left( - \sum\limits_{j=1}^{k} \Lambda _{j}(t) \right)}. \end{aligned}$$

The cumulative incidence function (CIF) of cause *k* is defined as $I_{k}(t) = Prob(T \le t, D = k)$, the probability of failing from cause *k* before time *t*. This can be linked to the cause-specific hazards through the expression:

4 $$\begin{aligned} I_{k}(t) = \int _{0}^{t} \lambda _{k}(s) S(s) ds. \end{aligned}$$

This is also called the subdistribution function based on the fact that the cumulative probability to fail from cause *k* cannot reach one, and therefore, it is not a proper probability distribution.

### Regression models for competing risks

#### Cause-specific Cox model

Regression on cause-specific hazards is an extension of the popular Cox proportional hazards model for CRs [10, 33]. The cause-specific hazard of cause *k* of a subject with covariate vector $\mathbf {Z}$ is modelled as

5 $$\begin{aligned} \lambda _{k}(t|\mathbf {Z}) = \lambda _{k, 0}(t) \exp {\left(\varvec{\beta }_{k}^{T} \mathbf {Z}\right)}, \end{aligned}$$

where $\lambda _{k, 0}(t)$ is the cause-specific hazard, and the vector $\varvec{\beta }_{k}$ represents the effects of covariates on cause *k*. Patients who move to another state other than *k* are censored at their transition time.

#### Fine and Gray model

In 1999, Fine and Gray introduced a subdistribution hazards model, which can directly regress on CIF [11]:

6 $$\begin{aligned} \tilde{\lambda }_{k}(t) = - \frac{d \log (1 - I_{k}(t))}{dt}. \end{aligned}$$

For the cause-specific Cox model, the risk set (number of patients at risk) decreases at each time point where there is a failure of another cause. On the other hand, for Fine and Gray’s model, individuals who fail from another cause remain in the risk set. The subdistribution hazards are then modelled assuming proportional hazards:

7 $$\begin{aligned} \tilde{\lambda }_{k}(t |\mathbf {Z}) = \tilde{\lambda }_{k, 0}(t) \exp {\left({\varvec{\beta }_{k}^{T}} \mathbf {Z}\right)}. \end{aligned}$$

Similar to the standard Cox model, the partial likelihood approach is used to estimate the parameters.

### Machine learning techniques for competing risks

#### Random survival forests

Random survival forests for competing risks (RSFCR) [25] are an extension of the RSF framework [21, 22] for CRs with right censored data proposed by Ishwaran et al. in 2014. It is a fully non-parametric ensemble tree approach for the estimation of the CIF for competing events (CIF and cause-specific hazard function are related as shown in equation (4)). RSFCR can directly model non-linear effects and interactions to perform accurate prediction without making any prior assumptions about the underlying data.

The algorithm of RSFCR is based on recursive binary partitioning while injecting randomness in two ways: (a) drawing *B* bootstrap samples from the learning data, and (b) growing a single CRs tree for each bootstrap sample by randomly selecting a subset of candidate variables at each node (region of the tree). A CR splitting rule is maximised to split each parent node into daughter nodes using the selected variables. The authors propose two splitting rules: either an event-specific or a combination of event-specific splitting rules across the *k* events. Here, the event-specific splitting rule was applied because disease progression was of major interest (weighted log-rank splitting, technical details in [25]). Then each tree is grown to full size under the constraint that terminal nodes (the ends of each tree) should have at least one unique case. In the terminal nodes, the Kaplan-Meier [8] and the Aalen-Johansen [34] methodologies are used to estimate the event-free survival function and the cause-specific CIF, respectively. Finally, the ensemble estimates are calculated averaging each estimator over the *B* grown trees. More technical details are provided in Additional file 2.

#### Partial logistic artificial neural networks

In 2006, Biganzoli et al. extended the partial logistic artificial neural network to competing risks (PLANNCR) for the joint modelling of discrete cause-specific hazards [18, 23]. PLANNCR is a feed-forward network comprised of a group of units called nodes (or neurons) in each layer. It has an input layer that picks up the signals and passes them to a single hidden layer after the application of an activation (also called transformation) function. An activation function modulates the degree of non-linearity transferred from the input features to the hidden layer. Connections between the artificial neurons of different layers are called edges - each having a weight. Weights are adjusted through training increasing or decreasing the strength of each connection [35]. Signals are transmitted towards the output layer, which provides a smoothed estimation of discrete conditional event probabilities (in multiple output nodes; each for an event), with another activation function.

For the purpose of implementation, survival times are discretized into a set of $l = 1, \cdots , L$ disjoint intervals $A_{l} = (\tau _{l-1}, \tau _{l}]$, where $0=\tau _{0}<\tau _{1}<\cdots <\tau _{L}$ is a set of pre-defined time points (usually years). For the $l^{th}$ interval, observed times are grouped on a single point $\tau _{l}$. Data has to be transformed into a longitudinal format where the time variable (in intervals) is added as part of the input features next to the prognostic features. Subjects are repeated for the number of intervals observed on the training data, and for all time intervals on the test data. PLANNCR can model non-linear, non-proportional, and non-additive effects between the prognostic factors on the cause-specific hazards. Here, without loss of generality, each subject was repeated for 1 up to 11 time intervals denoting years since surgery. The last interval included survival times longer than 10 years (subsequent intervals were not of interest).

In the CRs model, the response vector has $R + 1$ variables, with $r = 1, \cdots ,R$ the possible causes of interest (here $R = 2$). Let $\mathbf {z}_{k} = (\tau _{l}, \mathbf {x}_{k}$) be defined by two components: the covariate vector $\mathbf {x}_{k}$ ($k = 1, 2, \cdots , p$) and the time interval $\tau _{l}$. The joint dependence of the discrete cause-specific hazards is modelled as:

8 $$\begin{aligned} \eta _{lr}(\mathbf {z}_{k}, \varvec{\beta }) = \beta _{0} + \sum _{h=1}^{H} \beta _{r}^{a} \alpha _{h}\left(\beta _{0h} + \varvec{\beta }_{h}^{T}\mathbf {z}_{k}\right) \end{aligned}$$

where $h = 1, \cdots , H$ nodes in the hidden layer, $\varvec{\beta }$ the vector of estimated weights for the input-hidden ($\beta _{01}, \cdots , \beta _{0H}, \beta _{1}, \cdots , \beta _{H}$), hidden-output layers ($\beta _{0}, \beta _{1}^{a}, \cdots , \beta _{R}^{a}$), and $\alpha _{h}$ the sigmoid (logistic) activation function for the hidden layer $\alpha _{h}(\mathbf {z}_{k}, \varvec{\beta }_{h}) = \frac{\exp (\beta _{0h} + \beta _{h}^T \mathbf {z}_{k})}{1 + \exp (\beta _{0h} + \beta _{h}^T\mathbf {z}_{k})}$.

Activation function for the output layer is the softmax providing the discrete cause-specific hazards:

9 $$\begin{aligned} \tilde{h}_{lr}(\mathbf {z}_{k}, \varvec{\beta }) = \frac{\exp \big (\eta _{lr}(\mathbf {z}_{k}, \varvec{\beta })\big )}{\sum _{r = 1}^{R+1} \exp \big (\eta _{lr}(\mathbf {z}_{k}, \varvec{\beta })\big )}, \end{aligned}$$

for $l = 1, \cdots , L$ intervals, and $r = 1, \cdots ,R$ causes of interest. Since PLANNCR has a different output node for each CR (1 + *R* output nodes in total), it is an extension of standard neural networks for multiple classification resorting to the multinomial likelihood. For the rest of this paper, this will be called PLANNCR original [23].

Similar extensions to the specification of the PLANNCR are provided as in Kantidakis et al. (PLANN extended, 2020) [20]. More specifically, PLANNCR extended is tuned investigating two new activation functions for the hidden layer: (1) the rectified linear unit (ReLU) a common activation function, $\alpha _{h}(\mathbf {z}_{k}, \varvec{\beta }_{h}) = \max (0, \beta _{0h} + \beta _{h}^T \mathbf {z}_{k})$, or (2) the hyperbolic tangent (tanh), $\alpha _{h}(\mathbf {z}_{k}, \varvec{\beta }_{h}) = \frac{1 - \exp (-2(\beta _{0h} + \beta _{h}^T \mathbf {z}_{k}))}{1 + \exp (-2(\beta _{0h} + \beta _{h}^T \mathbf {z}_{k}))}$. Each time a neural network is fitted with one of these activation functions for the hidden layer or with the sigmoid (logistic) activation function (as in PLANNCR original). Note that the activation function for the output layer is necessarily the softmax to provide smoothed discrete hazard estimation. New hyperparameters are specified in a state-of-the-art R library [36]. In contrast with Kantidakis et al. (2020), the *L* non-overlapping intervals are specified in one time variable (instead of *L* separate variables) to not inflate the number of input features. Moreover, networks with two hidden layers are not tested here due to the danger for overfitting (small-medium sample size, small number of predictors). More technical details for PLANNCR original and PLANNCR extended are provided in Additional file 2.

### Model training

**Fig. 1:**
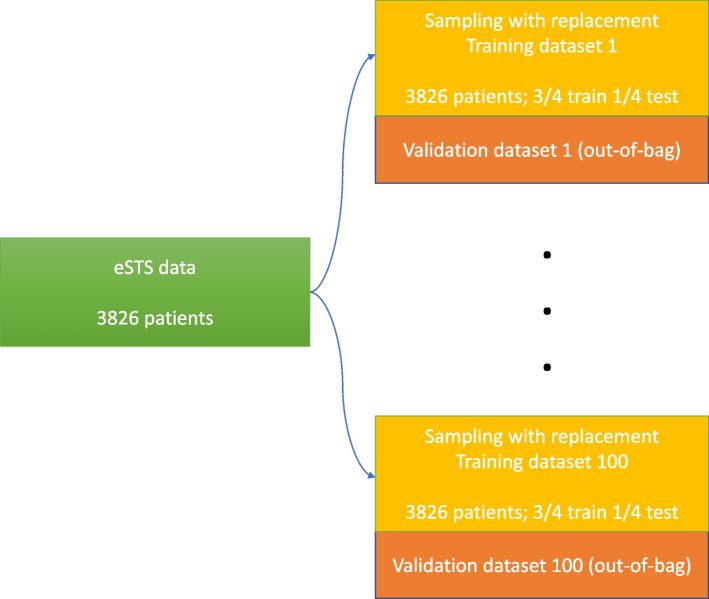
Illustration of the model training approach repeated 100 times. For the ML techniques, hyperparameters were tuned on the training datasets. Final performance for all models was assessed on the validation datasets (left out samples)

Figure 1 shows how model training was performed. Based on the original eSTS data, 100 bootstrapped training datasets were drawn with 3826 patients each (sampling with replacement, $\approx$ 63.2% of the original data). These datasets were randomly split into two complementary parts to tune the hyperparameters of the ML models using grid search ($\frac{3}{4}$ to train the models and $\frac{1}{4}$ to test their performance, same parts for all methods). Performance of the final models was assessed on the validation data, which were the left out samples (out-of-bag, $\approx$ 36.8% of the data). Out-of-bag error estimates are almost identical to *N*-fold cross-validation [37]. For the standard regression approaches, models were built on each complete training dataset (consisted of 3826 patients) using the nine covariates. Their predictive performance was evaluated on the respective validation dataset. Complex functional form dependencies (non-linear, non-additive, time-dependent effects) were not investigated. All analyses were performed in R programming language version 4.1.2 [26]. Packages used in the implementation and tuning parameters for the ML techniques are provided in Additional file 2.

### Predictive performance assessment

Predictive performance of the methods was assessed in terms of discrimination and calibration on each validation dataset. The Area Under the Curve (AUC) and Brier score with CRs were used. Miscalibration (absolute accuracy error) was also estimated. These evaluation measures were employed since they are model agnostic - they can be applied to any model to evaluate its predictive performance. Other measures such as the Akaike Information Criterion (AIC) or the Bayesian Information Criterion (BIC) were not selected, since they cannot be (easily) calculated for comparison of the different SM and ML techniques applied here.

Following Blanche et al. [38], we present the dynamic version of the measures with CRs (see also [39]). Let $\pi _{i}(\cdot ,\cdot )$ be a subject-i specific prediction process ($i = 1, 2, \cdots , n$ independent and identically distributed subjects) for all landmark times *s* (times at which predictions are made) and prediction horizon *t*. Without loss of generality, we set $\pi _{i} (s, t) = 0$ for all subjects *i* who are no longer at risk at time *s*, and focus on prediction of event $D = 1$ (main event investigated). A dynamic AUC at landmark time *s* for a prediction horizon *t* can be defined as

10 $$\begin{aligned} AUC(s, t) = Prob \Big (\pi _{i} (s, t)> \pi _{j} (s, t) | \Delta _{i}(s, t) = 1, \Delta _{j}(s, t) = 0, T_{i}> s, T_{j} > s \Big ), \end{aligned}$$

where $\Delta _{i} (s, t)$ = $\mathbbm {1}_{s<T_{i}\le s+t, D_{i} = 1}$, $\Delta _{i} (s, t) = 1$ when subject *i* experiences the main event of interest within time interval $(s, s + t]$ (case), and $\Delta _{i} (s, t) = 0$ when subject *i* experiences a competing event within the time interval or is event-free at $s+t$ (control) [40].

Dynamic AUC with CRs is a measure of discrimination. It typically ranges from 0.5 to 1 (the higher the better). A good predictive accuracy is provided by a model that usually gives higher predicted risks of event for subjects who experience the event of interest compared to subjects who did not experience the event of interest.

A more complete predictive accuracy measure with CRs is the Brier score. The dynamic expected Brier score can be written as

11 $$\begin{aligned} BS(s, t) = \mathbb{E} \left[ \left( \Delta (s, t) - \pi (s, t) \right)^{2} | T > s\right]. \end{aligned}$$

This expression can be expanded based on Graaf et al. 1999 [41] taking the following form

12 $$\begin{aligned} BS(s, t) = \mathbb{E} \left[ \left( \mathbb{E} [\Delta (s, t) | H(s) ] - \pi (s, t) \right)^{2} | T> s \right] + \mathbb {E} \left[ \big ( \Delta (s, t) - \mathbb{E} [\Delta (s, t) | H(s) ] \big )^{2} | T > s \right], \end{aligned}$$

where $H(s) = \{ \mathbf {X}, Y(s), T>s \}$ the information at time *s* used to compute the prediction of $\pi (s, t)$. The first term in (12) measures calibration - how close the predictions are to $\mathbb{E} [\Delta (s, t) | H(s)]$, the “true” underlying risk of event in $(s, s+t]$ given *H*(*s*). In addition, the second term depends on the discrimination ability of *H*(*s*). Thus, Brier score is a measure of both calibration and discrimination. Typically, it ranges from 0 to 0.25 (lower values mean smaller prediction error).

When censored data are present, the indicator $\Delta _{i}(s, t)$ is unknown (cannot be computed) for all subjects *i* censored within interval $(s, s+t]$. Therefore, the Inverse Probability of Censoring Weighting (IPCW) technique has to be applied for the estimation of both dynamic AUC and Brier score for CRs. For details see [38]. Here, the landmark time was set to $s = 0$ (baseline) for all analyses as all prognostic factors were time fixed.

Last, the predictive ability of the methods was evaluated based on their miscalibration on each validation dataset (see Fig. 1). Model calibration refers to the agreement between observed and predicted outcomes, in this case agreement between observed and predicted cumulative incidence event probabilities for a cause $D = k$ at time $t = t_{0}$ [42, 43]. For each SM and ML model, the predicted cumulative incidence event probabilities are estimated on a validation dataset, and the data is split into $m = 4$ equally sized groups based on the quantiles of the predicted event probabilities. Quantiles were selected instead of (for instance) deciles to avoid any computational issues. Then, the observed cumulative incidence probabilities are calculated for each group. Miscalibration is defined as the mean squared error (MSE) of the difference between the observed and the predicted cumulative probabilities of failure from a specific cause $D = k$ at time horizon $t = t_{0}$

13 $$\begin{aligned} MSE_{k}(t_{0}) = \frac{\sum _{m = 1}^{4} \left[ I_{k}^{(m)}(t_{0}) - \hat{I_{k}}^{(m)}(t_{0}) \right]^{2}}{4}, \end{aligned}$$

with $I_{k}^{(m)}(t_{0})$ and $\hat{I_{k}}^{(m)}(t_{0})$ the observed and predicted cumulative event probability for group *m*, respectively.

## Results

In this section, results for the eSTS data are presented. The following models are compared in terms of predictive performance: (1) Cause-specific Cox, (2) Fine-Gray, (3) PLANNCR original, (4) PLANNCR extended, (5) RSFCR. Each model is assessed on 100 validation datasets (see Fig. 1). More results about the comparison between the methods are provided in Additional file 3.

### PLANNCR tuned with Brier score or AUC at 5 years

The hyperparameters selected for PLANNCR original and PLANNCR extended are provided in section 1 of Additional file 3. The most effective combinations are reported separately based on the Brier score / AUC at 5 years (5-year horizon was of major clinical interest).

For PLANN original, both performance measures selected the same values for the 2 hyperparameters (*size* and *decay*). On the other hand, separate hyperparameters were selected for PLANNCR extended on a 5-D space (*nodesize*, *dropout rate*, *learning rate*, *momentum*, *weak class weight*). The technical details can be found in Additional file 2. From the 3 activation functions tested for the hidden layer (“sigmoid”, “relu”, “tanh”), the “sigmoid” provided the best performance on the training data for both Brier score and AUC. A *weak class weight* of 1 was selected (no adjustment for disease progression or death).

The performance of the tuned PLANNCR extended was compared for disease progression (event of interest). Results are presented in Table 2. PLANNCR extended tuned with Brier score at 5 years had a better performance in terms of Brier score and miscalibration at 2, 5, or 10 years. However, PLANNCR extended tuned with AUC at 5 years had a better performance regarding AUC at 5 and 10 years. These results were expected as Brier score is a more complete measure taking into account both discrimination and calibration. For the rest of the results presented below, optimal combinations for Brier score at 5 years were selected for PLANNCR extended.

**Table 2:** Mean predictive performance of PLANNCR extended for disease progression (event of interest) tuned with Brier score or AUC at 5 years. The 95% confidence intervals are provided in parentheses based on 100 validation datasets

Performance	PLANNCR extended with Brier score	PLANNCR extended with AUC
Brier score at 2 years	0.208 (0.198 - 0.220)	0.214 (0.201 - 0.226)
Brier score at 5 years	0.228 (0.221 - 0.235)	0.231 (0.225 - 0.236)
Brier score at 10 years	0.238 (0.229 - 0.247)	0.240 (0.234 - 0.247)
AUC at 2 years	0.661 (0.637 - 0.688)	0.659 (0.640 - 0.683)
AUC at 5 years	0.652 (0.612 - 0.689)	0.660 (0.633 - 0.685)
AUC at 10 years	0.629 (0.576 - 0.681)	0.631 (0.582 - 0.678)
Miscalibration at 2 years	0.008 (0.003 - 0.017)	0.013 (0.006 - 0.022)
Miscalibration at 5 years	0.003 (0.001 - 0.008)	0.008 (0.004 - 0.014)
Miscalibration at 10 years	0.002 (0.000 - 0.008)	0.004 (0.001 - 0.009)

### Predictive performance comparison

In this section, the five methods are compared on the 100 validation datasets for different predictive performance measures: (1) Brier scores, (ii) AUC, (iii) miscalibration at 2, 5, and 10 years, respectively, for disease progression (local recurrence or distant metastasis). Optimal hyperparameters and additional plots for the event of interest (disease progression) and the competing event (death) are included in sections 1 and 2 of Additional file 3.

#### Brier score - AUC

**Fig. 2:**
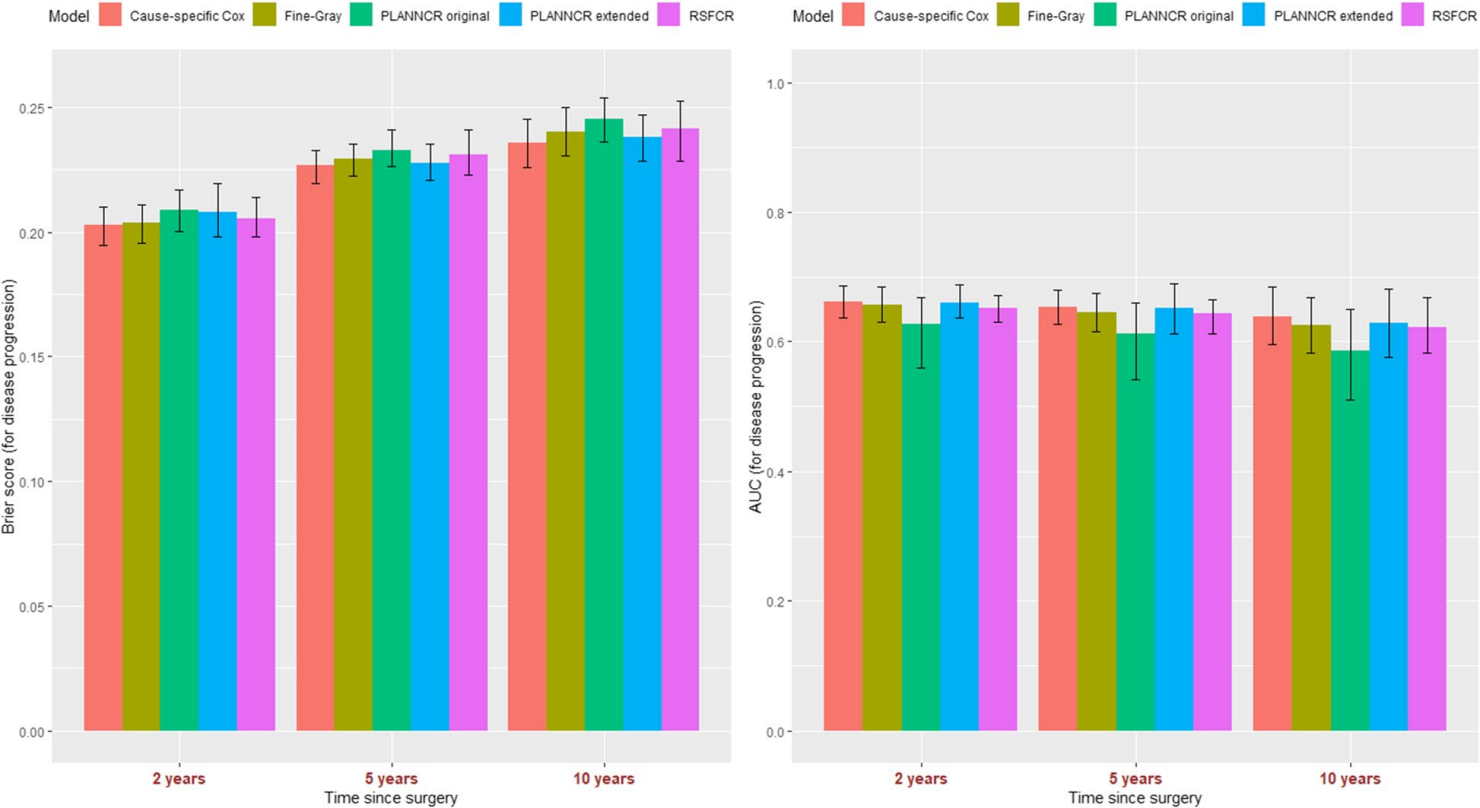
Predictive performance of cause-specific Cox model, Fine-Gray model, PLANNCR original, PLANNCR extended (tuned with Brier score at 5 years and including the “sigmoid” activation function for the hidden layer), and RSFCR for the event of interest: disease progression ± 95% percentile confidence intervals based on 100 validation datasets. Left panel: Brier score, right panel: AUC at 2, 5, and 10 years since surgery

Figure 2 shows the Brier score (lower values better) and AUC (higher values better) at 2, 5 and 10 years since surgery for all methods regarding disease progression.

For the time-dependent Brier score, the cause-specific Cox model had in general the best performance followed by the Fine-Gray model and RSFCR at 2 years, and the PLANNCR extended and Fine-Gray at 5 and 10 years. PLANNCR original had slightly the worst performance at these time points. 95% confidence intervals (CI) based on the percentile method for 100 validation datasets using the out-of-bag data overlapped. PLANNCR extended had marginally larger 95% CI at 2 years and RSFCR at 10 years. Regarding AUC at 2, 5, and 10 years, the cause-specific Cox model and the PLANNCR extended had the best performance (very close to each other) followed by Fine-Gray model, RSFCR and PLANNCR original in decreasing order of performance. The 95% confidence intervals were very similar for the methods, except for PLANNCR original which had much wider intervals at all times. This means that its discrimination ability (AUC) was not consistent (fluctuated) in the validation datasets.

Figure S1 in Additional file 3 provides the same plot with PLANNCR extended tuned with AUC at 5 years. The predictive ability decreased in terms of Brier score but slightly increased regarding AUC at 5 and 10 years (see also Table 2). Figures S3 and S5 in Additional file 3 illustrate the prognostic ability (Brier score, AUC) of all models for death (the competing event). The SM (cause-specific Cox and Fine-Gray) had the lowest Brier score followed by the RSFCR. PLANNCR models had worse performance and larger CI than the rest at 2 years. PLANNCR original continued to have larger CIs at 5 and 10 years, whereas PLANNCR extended had narrower CIs at 5 and 10 years (more consistent performance). For AUC, the cause-specific Cox model and the PLANNCR extended had the highest values followed by the Fine-Gray model and the RSFCR. PLANNCR original the lowest performance and the largest 95% CI.

#### Miscalibration

**Fig. 3:**
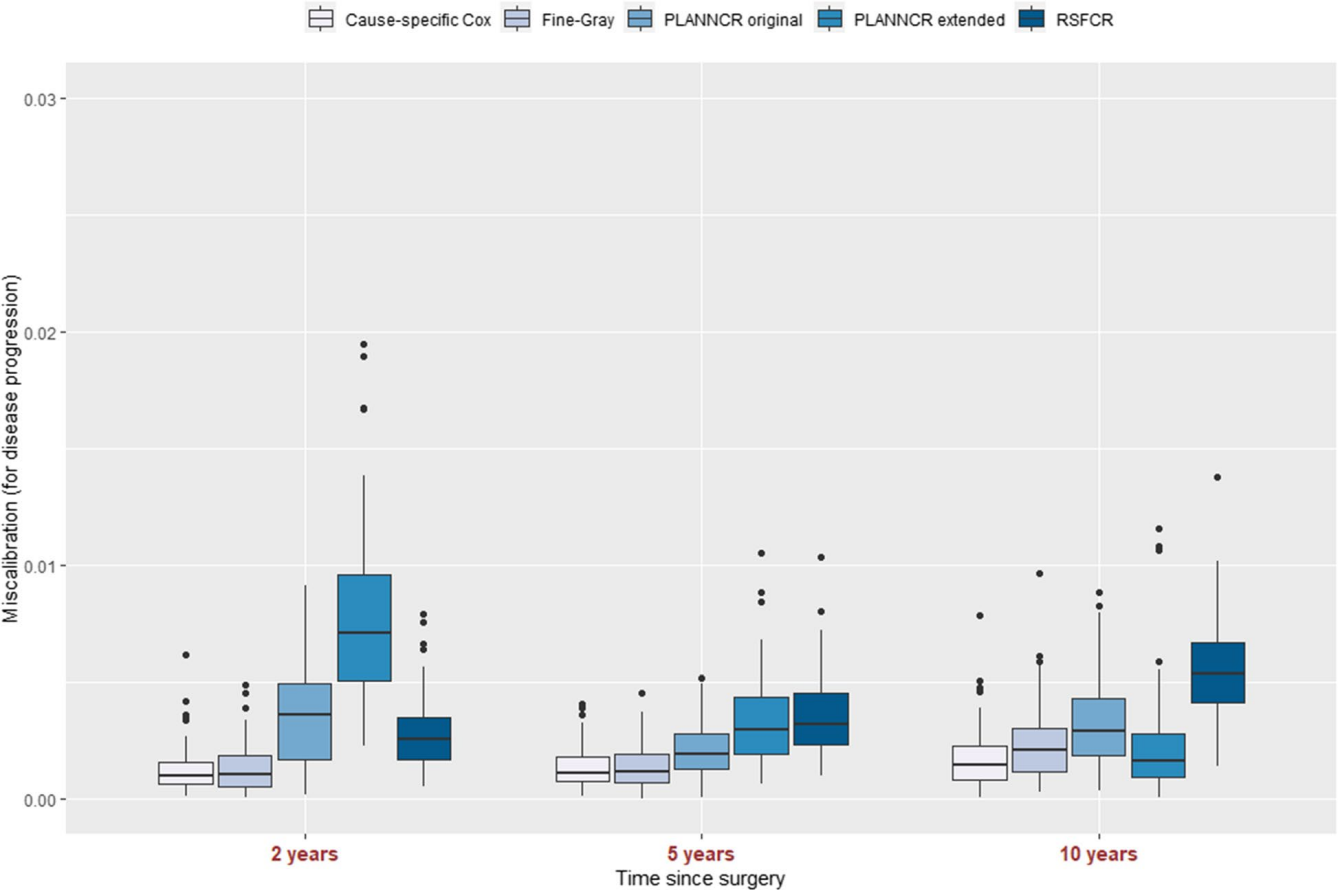
Miscalibration of cause-specific Cox model, Fine-Gray model, PLANNCR original, PLANNCR extended (tuned with Brier score at 5 years), and RSFCR at 2, 5, and 10 years for the event of interest: disease progression based on 100 validation datasets. Miscalibration was calculated as the mean squared error (MSE) between the observed and the predicted cumulative incidence event probabilities (for 4 groups)

The five models were investigated in terms of miscalibration (definition in section “Predictive performance assessment”) at 2, 5, and 10 years. Results are depicted in Fig. 3 with boxplots. The SM (cause-specific Cox model, Fine-Gray) had by far the lowest miscalibration error at 2 years for disease progression (cause 1). The SM and then the PLANNCR original had the lowest miscalibration at 5 years (the SM and PLANNCR extended at 10 years). PLANNCR extended had the highest miscalibration error at 2 years, the second highest at 5 years and the lowest at 10 years (next to cause-specific Cox model for this time point). The RSFCR had the worst calibration at 5 and 10 years for the cumulative incidence of the event of interest.

The miscalibration plot for PLANNCR extended tuned with AUC at 5 years is available in Additional file 3 (Fig. S2). PLANNCR extended is less well calibrated compared to Fig. 3. This result was expected since in the Supplementary figure the model was tuned for discrimination only (AUC at 5 years), whereas in Fig. 3 it was tuned taking into account both discrimination and calibration (Brier score at 5 years). Figures S4 and S6 show the miscalibration error for all five methods for the competing event (death). The cause-specific Cox model and the Fine-gray model had the lowest miscalibration error. RSFCR show a similar miscalibration error for death at 2 and 5 year and slightly worse error at 10 years. The two neural networks had the highest miscalibration error at any time point (distinct from the other three models). A tentative explanation of the higher PLANNCR miscalibration for the competing event is that it arises from heavier regularisation of the predicted death probabilities (for a given time point) resulting in a smaller spread of the predictions there. A solution to improve the calibration could be to tune the performance of PLANNCR (e.g. Brier score at 5 years) for the competing event. However, as disease progression was of major interest here, PLANNCR original and extended were both tuned for disease progression.

## Discussion

To the best of our knowledge, this is the first study which compared SM with ML techniques for CRs in soft-tissue sarcoma. A total of 3826 retrospectively collected patients were analysed with high-grade eSTS based on nine prognostic factors (small/medium sample size, low-dimensional setting). The SM (cause-specific Cox, Fine-Gray) and the RSFCR used exact times to event whereas the neural networks (PLANNCR original, PLANNCR extended) required a data preparation into a long format where the exact time points were turned into *L* separate time intervals (years). The five methods predicted the cumulative incidence of disease progression (event of interest) and death (competing event) since the date of surgery.

The results showed that the ML models have similar performance to the SM in terms of Brier score and AUC at 2, 5, and 10 years for disease progression and death (95% confidence intervals overlapped). Predictive ability of PLANNCR extended was usually better than RSFCR and PLANNCR original especially for AUC. This means that PLANNCR extended had the ability to discriminate better between low and high risk groups of patients. Nevertheless, the SM were frequently better calibrated than the three ML techniques. Miscalibration of PLANNCR original and extended was more pronounced for the competing event. These findings are consistent with a simulation study of our group that compared the predictive performance of SNN (PLANN original and extensions) with Cox models for osteosarcoma data in a similar simple setting (250 or 1000 patients, five prognostic factors) [44]. Hence, more attention to model calibration (absolute predictive accuracy) is urgently needed for ML methods.

For this work, we sampled with replacement 100 times (bootstrapping) from the eSTS data to train the ML models. Then, the left out samples were used to internally validate all models’ performance and obtain empirical 95% CIs (see Fig. 1). This can be an advantageous approach when the sample size is limited because it avoids decreasing the number of patients for model development / validation. However, it comes with a cost as this procedure is repeated multiple times and is therefore computationally expensive. The performance of all models was assessed with two time-dependent measures: Brier score (discrimination and calibration) and AUC (discrimination) at 2, 5, and 10 years, respectively. We chose the time-dependent AUC over the adaptation of Harrell’s concordance index to the CRs setting [45, 46] - a global performance measure for discrimination - since the latter is not a proper measure for the evaluation of *t*-year predicted risks (see [47]).

Two regression models for CRs were applied for the comparison with ML techniques; the cause-specific hazard regression Cox and the Fine-Gray. The cause-specific Cox model might be better suited for addressing etiological questions, whereas the Fine-Gray for estimating the clinical prognosis of patients - which was the aim here [3, 5, 48]. Nonetheless, both SM were employed for a more comprehensive approach, providing similar results, and outperforming the ML models in calibration. Complex functional dependencies such as non-linear and non-additive effects were not investigated, which shows how effective the SM can be in simple settings (with small/medium sample size and limited number of predictors) despite they assume additivity of effects and proportionality of hazards over time. On the other hand, ML methods may be very flexible (no a priori modelling assumptions), but usually require (very) large datasets to ensure small overfitting of their developed clinical prediction models [49, 50].

Other ML-driven models have been recently proposed for survival analysis with CRs and their prognostic ability was compared with typical benchmarks such as the cause-specific Cox, Fine-Gray, and RSFCR. In 2017, Alaa and van der Schaar [51] proposed a non-parametric Bayesian model to jointly assess a patient’s risk of multiple competing adverse events. The patient’s cause-specific survival times are modelled as a function of the covariates using deep multi-task Gaussian processes. Bellot and van der Schaar [52] developed in 2018 a tree-based Bayesian mixture model for CRs. They constructed a hierarchical Bayesian mixture model through multivariate random survival forests and evaluated the importance of variables for each cause. Recently, a deep neural network (multiple hidden layers) was employed by Nagpal et al. called deep survival machines [53]. This is a parametric methodology to jointly learn a common deep non-linear representation of the input features. This network estimates separately the event distribution for each CR. Note that for this project, we only specified shallow neural networks (1 hidden layer) to avoid excessive danger of overfitting in this simple setting.

Focusing on the practical utility, the two SM have the advantage compared to three ML techniques examined. The latter require a substantial implementation time for data preprocessing, tuning of the parameters, and are computationally more intensive to run (in terms of hours here). At the same time model optimisation of PLANNCR is a delicate task which requires robust numerical methods and skillful use, else the network might converge in suboptimal minima in the error function [35]. From the three ML techniques, PLANNCR extended demanded more time and effort for training because of the larger number of tuning parameters (five versus two for PLANNCR original and RSFCR). On the contrary, the cause-specific Cox and Fine-Gray models do not require any hyperparameter tuning and offer a fast implementation.

Nowadays, the employment of ML is overhyped in some contexts of medicine due to the increased interest in applying modern techniques to create prediction models. Therefore, it is necessary to report prediction models powered by artificial intelligence completely and transparently to allow critical appraisal, reproducibility of the modelling steps and results by a wider audience, and to avoid research waste [14, 15, 54]. In general, a traditional regression approach may still provide more accurate predicted survival probabilities and prognostic performance compared to a state-of-the-art ML model, especially in non-complex medical settings (low-medium sample size, small number of predictors). In this instance, application of ML algorithms should only be motivated for exploration of the collected data.

In the future, it might be useful to compare the predictive ability of the cause-specific proportional hazard Cox model with the PLANNCR original / extended for time-dependent variables. The first method allows the inclusion of time-dependent covariates in standard software, and the second can naturally incorporate time-dependent covariates due to the essential data transformation into a long format for each patient. Moreover, the Fine-Gray and RSFCR can be extended to provide dynamic predictions with time-dependent covariates for CRs by creating a landmark dataset at a set of landmark time points $t_{LM}$ [55]. Last but not least, it would be interesting to compare the SM and ML techniques regarding interpretation. Overall, SM offer a more straightforward interpretation via cause-specific hazard ratios, while PLANNCR can provide the shape of the cause-specific hazard function over time and covariates, and RSFCR the variable importance. Further research is needed on a common metric to directly compare all methods.

## Conclusions

In this article, we discussed ML alternatives (PLANNCR original, PLANNCR extended, RSFCR) to SM (cause-specific Cox model, Fine-Gray) to build prognostic models for survival analysis with CRs in eSTS data with small/medium sample size and limited number of predictors (simple setting). Methods were compared in terms of discrimination and calibration. ML models reached an equivalent performance in terms of suitable predictive performance measures at 2, 5, or 10 years since surgery (95% confidence intervals overlapped), but the conventional regression models were generally better calibrated. Hence, more attention to calibration is needed. Modern ML-driven techniques are less practical as they require substantial implementation time (data preprocessing, hyperparameter tuning, computational intensity), whereas regression models are straightforward to use and can perform well without the additional workload of model training. Overall, complete and transparent reporting of all methods is required to allow critical appraisal, reproducibility, and avoid research waste. In our opinion, for non-complex real life data such as this, ML techniques should only be employed complementary to SM as exploratory tools of model’s performance.

## Supplementary Information

**Additional file 1.**

**Additional file 2.**

**Additional file 3.**

## Data Availability

The clinical data used for this research project is private. The R-code developed to perform this analysis is provided in the following GitHub repository https://github.com/GKantidakis/SM-vs-ML-for-CRs. The reader will also find a zip file with R-codes, which is a comprehensive example of this analysis in publicly available R data for Follicular Cell Lymphoma (data “follic”). The analysis plots of the “follic” data (n = 541, p = 4), which illustrate the same methodologies within non-complex data, support the findings of the eSTS data.

## Abbreviations

AUC: Area Under the Curve
CI: Confidence Interval
CIF: Cumulative Incidence Function
CRs: Competing risks
eSTS: Extremity soft-tissue sarcomas
IPCW: Inverse Probability of Censoring Weighting
KM: Kaplan-Meier
ML: Machine learning
MSE: Mean squared error
PLANN: Partial logistic artificial neural network
PLANN-ARD: Partial logistic artificial neural network - automatic relevance determination
PLANNCR: Partial logistic artificial neural network for competing risks
PLANNCR-ARD: Partial logistic artificial neural network for competing risks - automatic relevance determination
ReLU: Rectified linear unit
RSF: Random survival forests
RSFCR: Random survival forests for competing risks
SM: Statistical models
SNNs: Survival neural networks
