# Supplementary material for: Statistical models versus machine learning for competing risks: development and validation of prognostic models
Source: BMC Med Res Methodol. 2023 Feb 24;23:51. doi: 10.1186/s12874-023-01866-z (PMC9951458; doi:10.1186/s12874-023-01866-z)
Supplement: Supplementary file 1 — Additional file 1. [file 12874_2023_1866_MOESM1_ESM.pdf]

Original eSTS data

**Characteristics**	**Total** (N = 3826)
Gender (%)	
Female	1680 (43.91%)
Male	2011 (52.56%)
Missing	135 (3.53%)
Mean age in years (sd)	59.40 (18.10)
Mean tumor size in cm (sd)	9.04 (5.77)
Surgical margin (%)	
R_0	3028 (79.14%)
R_{1-2}	515 (13.46%)
Missing	283 (7.40%)
Adjuvant chemotherapy (%)	
No	3189 (83.35%)
Yes	470 (12.28%)
Missing	167 (4.36%)
Tumor grade (%)	
II	639 (16.70%)
III	3111 (81.31%)
Missing	76 (1.99%)
Histological subtype (%)	
Myxofibrosarcoma	689 (18.01%)
Synovial sarcoma	411 (10.74%)
MFH/UPS/NOS	1204 (31.47%)
Leiomyosarcoma	368 (9.62%)
Liposarcoma	388 (10.14%)
Other	452 (11.81%)
Missing	314 (8.21%)
Tumor depth (%)	
Superficial	912 (23.84%)
Deep	2493 (65.16%)
Missing	421 (11.00%)
Radiotherapy (%)	
No	1331 (34.79%)
Neoadjuvant	517 (13.51%)
Adjuvant	1878 (49.09%)
Missing	100 (2.61%)

Table S1: **Patient demographics before the imputation.** sd, standard deviation; R_0, negative margin; R_{1-2}, positive margin with tumor cells in the inked surface of the resection margin; MFH/UPS/NOS, alignant fibrous histiocytoma / undifferentiated pleomorphic sarcoma / (pleomorphic) soft tissue sarcomas not-otherwise-specified; histology ”Other”, angiosarcoma, clear cell sarcoma, conventional fibrosarcoma, epithelioid sarcoma, giant cell sarcoma, malignant granular cell tumor, malignant peripheral nerve sheath tumor, rhabdomyosarcoma (adult form), spindle cell sarcoma, unclassified soft tissue sarcoma and undifferentiated sarcoma.
