# Supplementary material for: Statistical models versus machine learning for competing risks: development and validation of prognostic models
Source: BMC Med Res Methodol. 2023 Feb 24;23:51. doi: 10.1186/s12874-023-01866-z (PMC9951458; doi:10.1186/s12874-023-01866-z)
Supplement: Supplementary file 2 — Additional file 2. [file 12874_2023_1866_MOESM2_ESM.pdf]

Technical details for the ML techniques

RSFCR

For RSFCR [1,2], the `randomForestSRC` package of the R programming language was used [3]. Tuning of the hyperparameters was done using grid search. The choice of the hyperparameters and their range (grid search) was provided based on recommendations by the authors of `randomForestSRC` [4]. Parameters tuned were `mtry` the number of candidate variables examined at each split point (range 1 - 5) and `nodesize` the average number of observations in the terminal nodes across the forest (range 10 - 30). Parameter `ntree` the number of bootstrapped trees grown was set to 1000 trees for a stable performance. Parameter `nsplit` the number of split points at which an X variable is tested using the ”logrank” splitting rule (cause of interest was disease-progression) was set to 2 to avoid bias towards the continuous predictive factors [2].

In general, parameters `ntree` and `mtry` are the most fundamental for RSFCR. The parameter `ntree` modulates the consistency of the forest's performance and `mtry` controls an important part of randomness during the growth of decision trees. Parameter `nsplit` with `nsplit` > 0 can be used to trigger a randomised selection of exactly nsplit points for each of the mtry variables within a node h. Last, parameter `nodesize` plays an important role in the topology of the trees as it controls the average node size of the forest. Large values in `nodesize` parameter will essentially force the forest to undergrow whereas small values will lead each tree to keep growing on with more and more noisy variables being selected. The best combination of parameters was determined based on the error of the forests on the test set (of the training datasets) defined as $E = 1 - C$, where C is an adaptation to Harrell's concordance index to the competing risks setting [5,6].

PLANNCR

PLANNCR is an extension of PLANN [7] and standard neural networks for multiple classification resorting to the multinomial likelihood. A data transformation to longitudinal format was required. The time interval was added next to the other input features to estimate smoothed conditional event probabilities for each event (alive/censored, disease progression, or death). Variables were presented in dummy coding - categorical variables as indicators and continuous variables standardised. Here, without loss of generality, each subject was repeated for 1 up to 11 time intervals denoting years since surgery. The last interval included survival times longer than 10 years (subsequent intervals were not of interest). Tuning of the hyper-parameters was done using grid search. The best combination of the parameters was determined on the test set of the training datasets based on either the time-dependent area under the curve (AUC) at 5 years, or the Brier score at 5 years (time-point of major clinical interest for disease progression of the eSTS patients) [8,9].

PLANNCR original

For PLANNCR original, the `nnet` package [10] of the R programming language was used. The choice of hyperparameters and their range (for the grid search) were based on reasoning from the original article by Biganzoli *et al.* [11]. Optimization was done via the BFGS method (quasi-Newton algorithm) of optim R function. Parameters tuned were i) `size` the number of nodes (units) in the hidden layer which determines the number of weights (values 2, 3, 4, $\cdots$, 14) and ii) `decay` a regularization technique applied to the error (loss) function which penalizes large weight values to avoid overfitting as $E^* = E + \lambda \sum w^2$ (values 0.01, 0.05, 0.1, 0.2, 0.3, 0.4 and 0.5). Having 15 inputs in total (14 prognostic variable levels in dummy coding + time interval variable) the optimal value for `size` is somewhere in the range 2-14.

PLANNCR extended

For PLANNCR extended, model tuning was performed in R with the package `keras` [12], which is an interface for the original state-of-the-art neural network library written in Python programming language. `keras` runs on top of `tensorflow` [13], which is a symbolic maths library used for ML. Two of the main advantages of this package are that it allows the use of distributed training of deep learning models on clusters of graphic processing units and the specification of many building blocks such as activation functions, layers, objectives, optimisers. Optimization was done with the stochastic gradient descent algorithm of `keras` (works better for shallow neural networks). To narrow down the grid of point combinations, search for training data was performed on a 5-D space of some of the most fundamental hyper-parameters. Those are `nodesize` the size of nodes in the hidden layer (values $2, 4, 6, \cdots, 14$), `dropout rate` that randomly selects the amount of nodes to be dropped-out with a given probability (values 0.1, 0.2 or 0.4), `learning rate` which is the step size of weight iteration (values 0.1, 0.2 or 0.4), `momentum` which helps to accelerate gradient vectors (values 0.8 or 0.9) and `weak class weight` that defines the weight of disease progression or death (values 1 or 1.25).

`Nodesize` defines the number of weights of the network and consequently the amount of its complexity. Having 15 inputs in total for the main analyses (14 prognostic variable levels in dummy coding + 1 variable for yearly time intervals), the optimal `nodesize` was somewhere in the range 2 - 14. `Dropout rate` is a technique used to control over-fitting [14]. Regarding the rest of the parameters, `learning rate` adjusts how fast the stochastic gradient descent iterative method uses stochastic approximation. `momentum` can accelerate the stochastic gradient vectors in the right directions and `weak class weight` can be used for re-weighting unbalanced classes (a small re-weighting adjustment was used). Additionally, we used early stopping to prevent over-fitting (3 epochs tolerance). We specified 20 training epochs and terminated training once the performance stopped improving on the validation set. Overall, training PLANNCR extended was more computationally intensive than PLANNCR original because of the increased number of hyperparameters. Moreover, it is worth mentioning that the implementation of PLANNCR extended within a modern library does not necessarily imply a better performance than PLANNCR original regarding the numerical optimization.

References

[1] Hemant Ishwaran, Udaya B. Kogalur, Eugene H. Blackstone, and Michael S. Lauer. Random survival forests. *The Annals of Applied Statistics*, 2(3):841–860, 2008. URL: https://projecteuclid.org/journals/annals-of-applied-statistics/volume-2/issue-3/Random-survival-forests/10.1214/08-AOAS169.short, doi:10.1214/08-AOAS169.

[2] Hemant Ishwaran, Thomas A. Gerds, Udaya B. Kogalur, Richard D. Moore, Stephen J. Gange, and Bryan M. Lau. Random survival forests for competing risks. *Biostatistics*, 15(4):757–773, 2014. doi:10.1093/biostatistics/kxu010.

[3] H. Ishwaran and U.B. Kogalur. Fast Unified Random Forests for Survival, Regression, and Classification (RF-SRC), 2022. URL: https://cran.r-project.org/package=randomForestSRC.

[4] Fast Unified Random Forests for Survival, Regression, and Classification (RF-SRC) • Fast Unified Forests with randomForestSRC. URL: https://luminwin.github.io/randomForestSRC/.

[5] Marcel Wolbers, Michael T. Koller, Jacqueline C.M. Witteman, and Ewout W. Steyerberg. Prognostic models with competing risks methods and application to coronary risk prediction. *Epidemiology*, 20(4):555–561, 2009. doi:10.1097/EDE.0b013e3181a39056.

[6] Marcel Wolbers, Paul Blanche, Michael T. Koller, Jacqueline C.M. Witteman, and Thomas A. Gerds. Concordance for prognostic models with competing risks. *Biostatistics*, 15(3):526–539, 2014. doi:10.1093/biostatistics/kxt059.

[7] E Biganzoli, P Boracchi, L Mariani, and E Marubini. Feed forward neural networks for the analysis of censored survival data: a partial logistic regression approach. *Statistics in medicine*, 17(10):1169–1186, 1998. doi:10.1002/(sici)1097-0258(19980530)17:10<1169::aid-sim796>3.0.co;2-d.

[8] Paul Blanche, Jean François Dartigues, and Hélène Jacqmin-Gadda. Estimating and comparing time-dependent areas under receiver operating characteristic curves for censored event times with competing risks. *Statistics in Medicine*, 32(30):5381–5397, 2013. doi:10.1002/sim.5958.

[9] Paul Blanche, Cécile Proust-Lima, Lucie Loubère, Claudine Berr, Jean François Dartigues, and Hélène Jacqmin-Gadda. Quantifying and comparing dynamic predictive accuracy of joint models for longitudinal marker and time-to-event in presence of censoring and competing risks. *Biometrics*, 71(1):102–113, 2015. doi:10.1111/biom.12232.

[10] Brian Ripley and William Venables. nnet: Feed-Forward Neural Networks and Multinomial Log-Linear Models, 2016. URL: https://cran.r-project.org/web/packages/nnet/index.html.

[11] Elia Biganzoli, Patrizia Boracchi, Federico Ambrogi, and Ettore Marubini. Artificial neural network for the joint modelling of discrete cause-specific hazards. *Artificial Intelligence in Medicine*, 37(2):119–130, 2006. doi:10.1016/j.artmed.2006.01.004.

[12] François Chollet. keras, 2015. URL: https://github.com/keras-team/keras.

[13] Martin Abadi, Paul Barham, Jianmin Chen, Zhifeng Chen, Andy Davis, Jeffrey Dean, Matthieu Devin, Sanjay Ghemawat, Geoffrey Irving, Michael Isard, Manjunath Kudlur, Josh Levenberg, Rajat Monga, Sherry Moore, Derek G Murray, Benoit Steiner, Paul Tucker, Vijay Vasudevan, Pete Warden, Martin Wicke, Yuan Yu, and Xiaoqiang Zheng. TensorFlow: A system for large-scale machine learning. In *12th {USENIX} Symposium on Operating Systems Design and Implementation ({OSDI} 16)*, pages 265–283, 2016. URL: https://ai.google/research/pubs/pub45381.

[14] Nitish Srivastava, Geoffrey Hinton, Alex Krizhevsky, Ilya Sutskever, and Ruslan Salakhutdinov. Dropout: A Simple Way to Prevent Neural Networks from Overfitting. *The Journal of Machine Learning Research*, 15(1):1929–1958, 2014. URL: http://jmlr.org/papers/v15/srivastava14a.html.
