# Supplementary material for: Statistical models versus machine learning for competing risks: development and validation of prognostic models
Source: BMC Med Res Methodol. 2023 Feb 24;23:51. doi: 10.1186/s12874-023-01866-z (PMC9951458; doi:10.1186/s12874-023-01866-z)
Supplement: Supplementary file 3 — Additional file 3. [file 12874_2023_1866_MOESM3_ESM.docx]

**Supplementary file 3 – Results**

# Hyperparameters selected for the ML techniques

Below, the optimal combinations are provided in green.

## a) For PLANNCR original

Tuned with Brier score / AUC at 5 years on training data (event of interest disease progression)

*nodesize* = 3, *decay* = 0.5 (Brier 5y = 0.229, AUC 5y = 0.631).

## b) For PLANNCR extended

(i) Tuned with Brier score at 5 years on training data (event of interest disease progression)

For activation function “sigmoid”:

*nodesize* = 12, *dropout* = 0.1, *learning rate* = 0.1, *momentum* = 0.8, *weak class* = 1 (Brier 5y = 0.227).

For activation function “relu”:

*nodesize* = 10, *dropout* = 0.4, *learning rate* = 0.1, *momentum* = 0.8, *weak class* = 1 (Brier 5y = 0.230).

For activation function “tanh”:

*nodesize* = 4, *dropout* = 0.1, *learning rate* = 0.1, *momentum* = 0.8, *weak class* = 1 (Brier 5y = 0.230).

(ii) Tuned with AUC at 5 years on training data (event of interest disease progression)

For activation function “sigmoid”:

*nodesize* = 6, *dropout* = 0.4, *learning rate* = 0.1, *momentum* = 0.8, *weak class* = 1 (AUC 5y = 0.662).

For activation function “relu”:

*nodesize* = 6, *dropout* = 0.4, *learning rate* = 0.1, *momentum* = 0.8, *weak class* = 1 (AUC 5y = 0.649).

For activation function “tanh”:

*nodesize* = 4, *dropout* = 0.4, *learning rate* = 0.1, *momentum* = 0.8, *weak class* = 1 (AUC 5y = 0.653).

## c) For RSFCR

Tuned with forest error on training data (event of interest disease progression)

Default parameters: *Ntree* = 1000, *nsplit* = 2, *splitrule* = “logrank”

Tuned parameters: *mtry* = 5, *nodesize* = 10 (Error = 0.276).

# Comparison of the predictive performance

## For disease progression (event of interest)


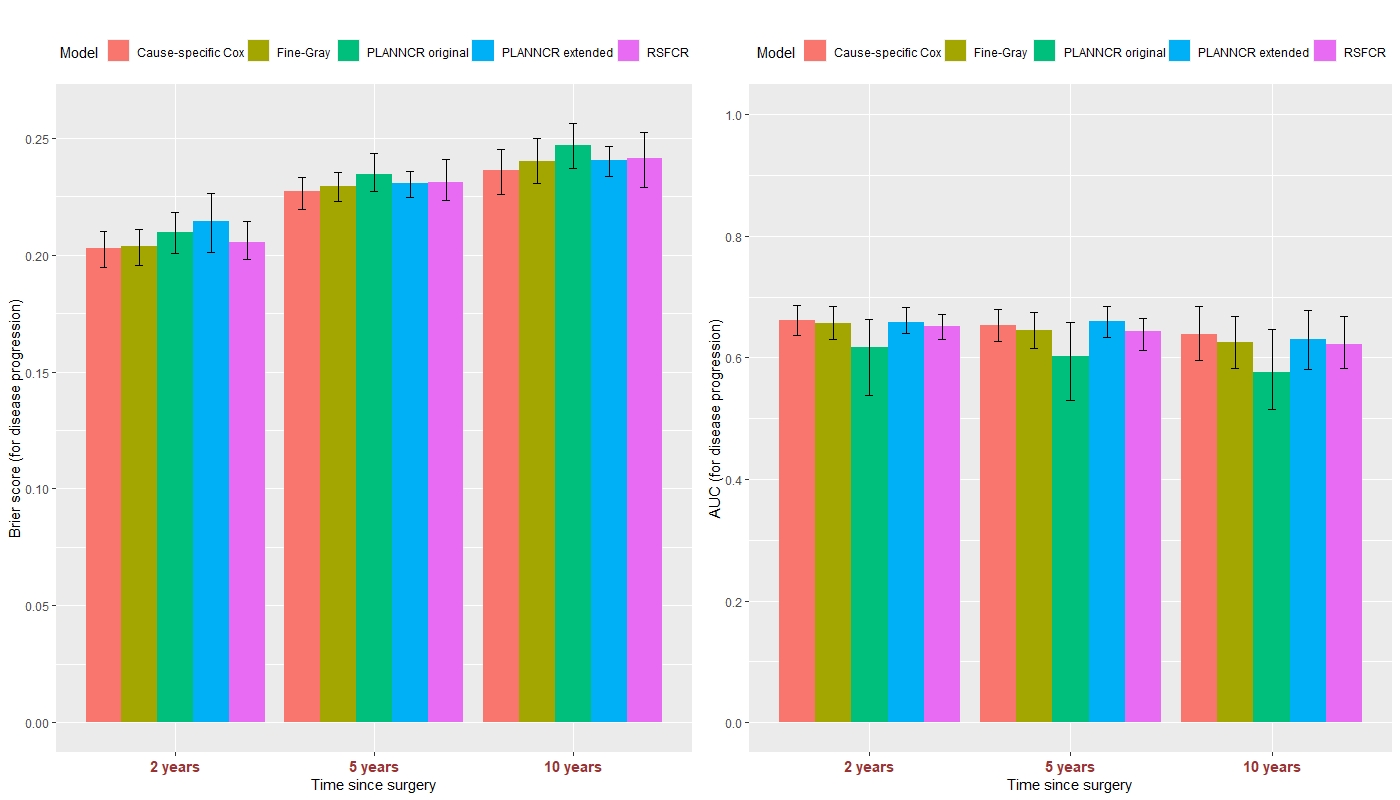


Figure S1: Predictive performance of cause-specific Cox model, Fine-Gray model, PLANNCR original, PLANNCR extended (tuned with AUC at 5 years), and RSFCR for the event of interest: disease progression ± 95% percentile confidence intervals based on 100 validation datasets. Left panel: Brier score, right panel: AUC at 2, 5, and 10 years since surgery.


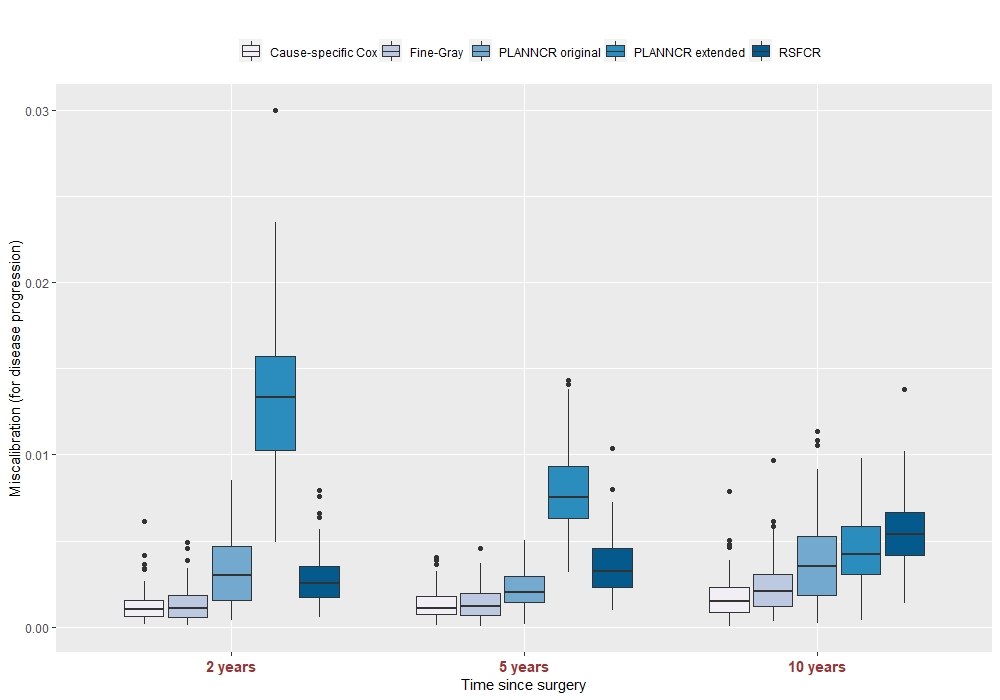


Figure S2: Miscalibration of cause-specific Cox model, Fine-Gray model, PLANNCR original, PLANNCR extended (tuned with AUC at 5 years), and RSFCR at 2, 5, and 10 years for the event of interest: disease progression based on 100 validation datasets. Miscalibration was calculated as the MSE between the observed and the predicted cumulative incidence event probabilities (for 4 groups).

## For death (competing event)


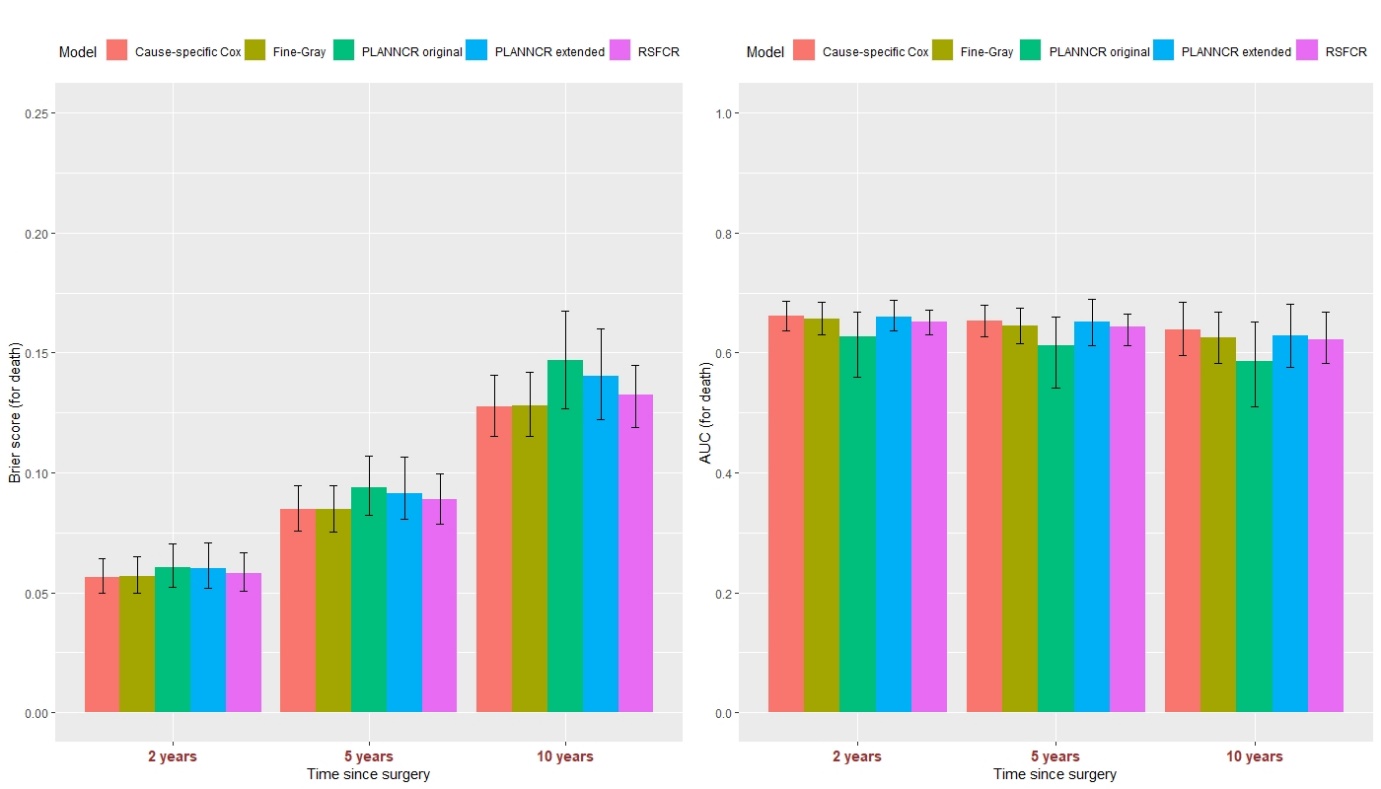


Figure S3: Predictive performance of cause-specific Cox model, Fine-Gray model, PLANNCR original, PLANNCR extended (tuned with Brier score at 5 years), and RSFCR for the competing event: death ± 95% percentile confidence intervals based on 100 validation datasets. Left panel: Brier score, right panel: AUC at 2, 5, and 10 years since surgery.


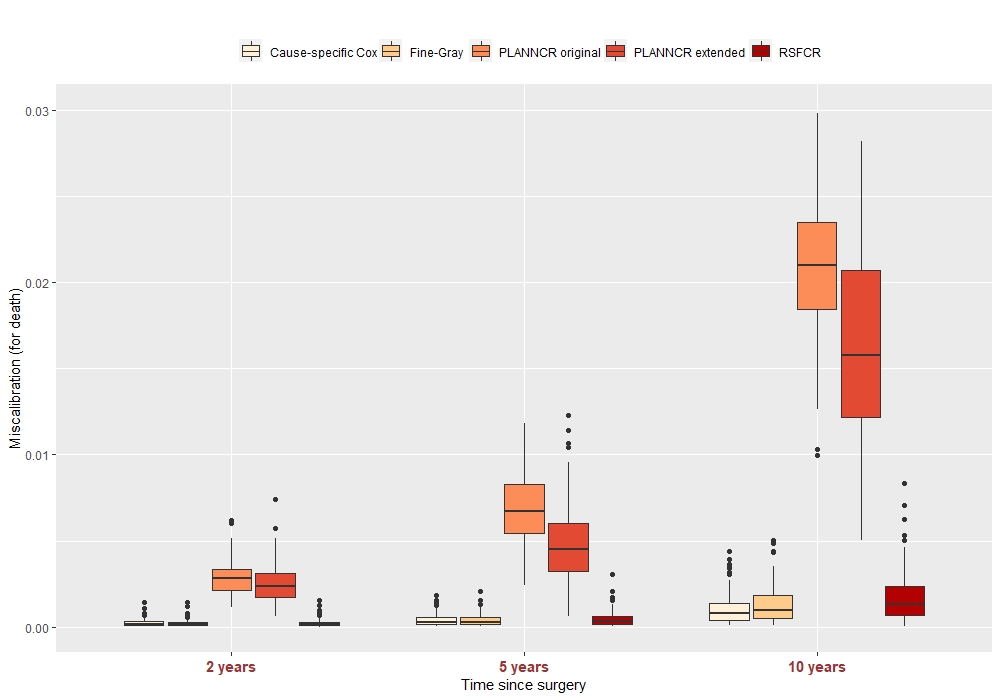


Figure S4: Miscalibration of cause-specific Cox model, Fine-Gray model, PLANNCR original, PLANNCR extended (tuned with Brier score at 5 years), and RSFCR at 2, 5, and 10 years for the competing event: death based on 100 validation datasets. Miscalibration was calculated as the MSE between the observed and the predicted cumulative incidence event probabilities (for 4 groups).


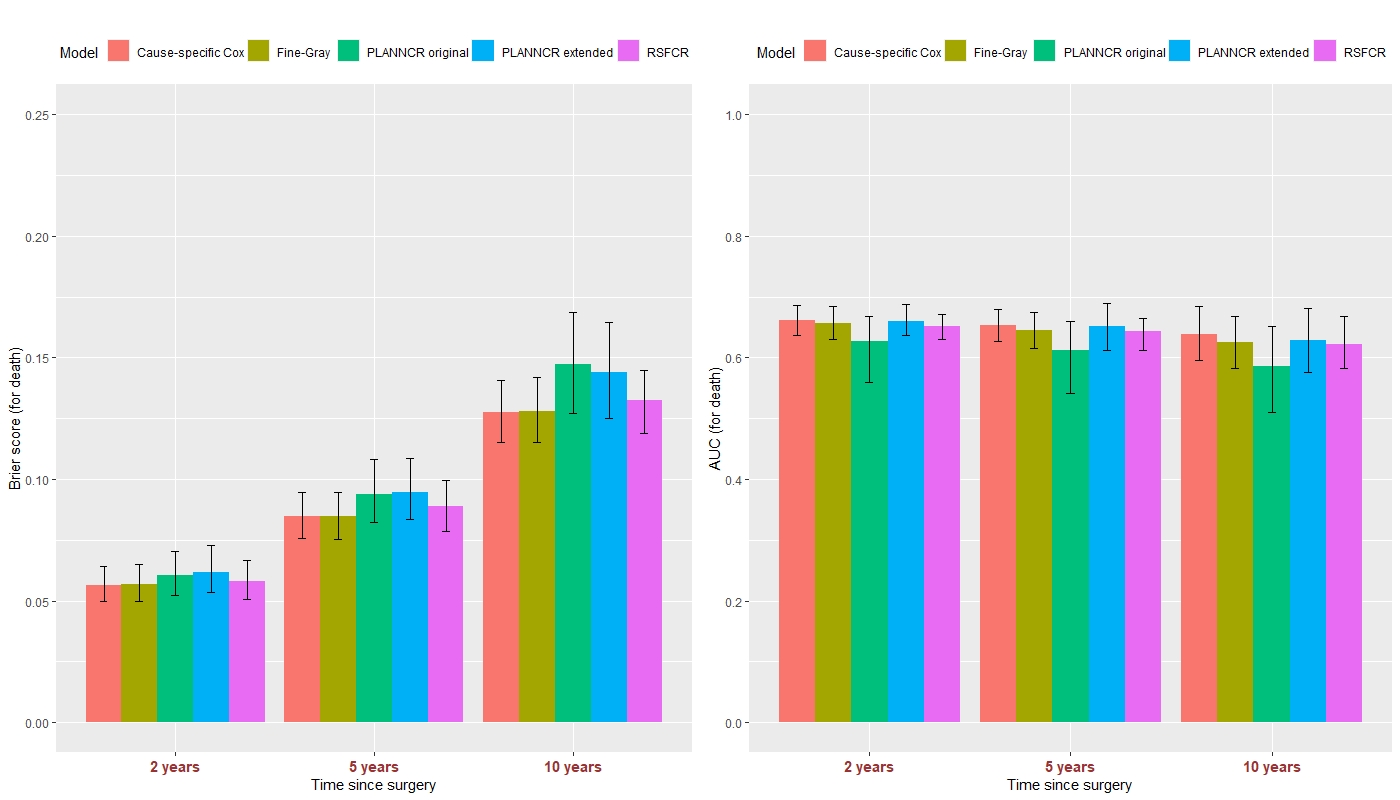


Figure S5: Predictive performance of cause-specific Cox model, Fine-Gray model, PLANNCR original, PLANNCR extended (tuned with AUC at 5 years), and RSFCR for the competing event: death ± 95% percentile confidence intervals based on 100 validation datasets. Left panel: Brier score, right panel: AUC at 2, 5, and 10 years since surgery.


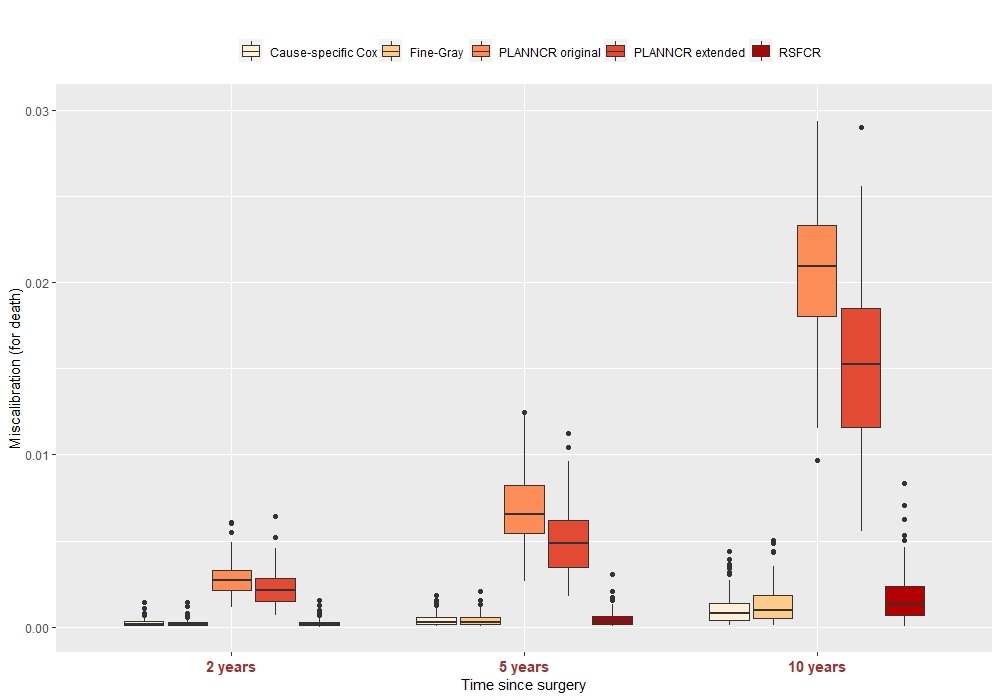


Figure S6: Miscalibration of cause-specific Cox model, Fine-Gray model, PLANNCR original, PLANNCR extended (tuned with AUC at 5 years), and RSFCR at 2, 5, and 10 years for the competing event: death based on 100 validation datasets. Miscalibration was calculated as the MSE between the observed and the predicted cumulative incidence event probabilities (for 4 groups).
